# Carl Gustav Jung: Paradigms and extra-terrestrial intelligence

**DOI:** 10.6026/9732063002001524

**Published:** 2024-11-30

**Authors:** Paul Shapshak

**Affiliations:** 1 Department of Internal Medicine, Morsani College of Medicine, Tampa, Florida 33606, USA

**Keywords:** Carl gustav jung, psychiatry, psychology, paradigm, collective unconscious, archetypes, individuation, shadow, anima and animus, synchronicity, self, persona, complexes, psychological types, non-verbal communication, extra-terrestrial intelligence (ETI), inter-stellar, inter-galactic, electromagnetic (light), neutrinos and communications

## Abstract

Carl Gustav Jung (1875-1961) developed multiple paradigms across personal, social and cultural domains. This report uses some of Jung's work across a broad context - the basis for possible communications with and among extra-terrestrial intelligences (ETIs). Jung's domains, among many, include collective unconscious, archetypes, individuation, shadow, anima and animus, synchronicity, self, persona, complexes and psychological types. This paper hypothesizes the applicability of Jung's paradigms and domains to communicate with ETI.

## Background:

Many key concepts Jung formulated include the collective unconscious, archetypes, individuation, shadows, anima/animus, synchronicity, self, persona, complexes and psychological types [1, 2]. These concepts in analytical psychology emphasize the diverse aspects of the psyche, internally and externally. This paper applies these concepts to ETI as a basis for communication.

## Collective unconscious:

Jung introduced concepts of the collective unconscious as unconscious mental concepts shared by all people across individuals, societies, cultures, civilizations and ethnicities. The collective unconscious is proposed as inherited and universal and that it is not a product of personal experience but a structure that is passed down biologically, containing universal elements common to all humanity [2, 3].

## Archetypes:

Archetypes are universal innate symbols, images, patterns and themes that emerge from the collective unconscious and often represented in myths, art and literature. Examples include the Hero, Mother, Shadow and Self. Such perceptions influence both individual psyches and cultural mythologies. Jung also stated that archetypes are templates for human thought and behavior and transcend cultural; boundaries [3, 4].

## Individuation:

The process of individuation is the integration of the conscious and unconscious mind, leading to self-realization and psychological maturity. This is a lifelong drive, which allows individuals to attain their true selves, distinct from societal expectations [5, 6].

## Shadow:

The Shadow represents a hidden part of the psyche, which includes repressed weaknesses, desires and instincts. Jung considered that integrating the Shadow into one's conscious awareness was crucial for personal development [7].

## Anima and animus:

These represent the feminine aspects of the male psyche (anima) and the masculine aspects of the female psyche (animus). Jung contended that achieving balance between these aspects was key to psychological wholeness [5].

## Synchronicity:

Jung developed the concept of synchronicity to explain meaningful coincidental events or occurrences, which appear connected by significance and connotation rather than by causality. He saw these events as expressions of universal underlying configurations. Jung directly addresses this in conjunction with Wolfgang Pauli, one of the founders of quantum mechanics. Possibly the quantum mechanics superposition principle relates to the underlying configurations addressed by Jung [1, 8].

## Self:

The Self, according to Jung, is the central archetype that represents the totality of the psyche, encompassing both the conscious and the unconscious. It is the lifelong goal of individuation and symbolizes unity and completeness [7].

## Persona:

The persona is the outward face individual's presents to the world, shaped by social roles, expectations and norms. Jung held that over-identification with the persona could lead to disconnects from the true self, impeding personal growth. Balancing the persona with the inner self is critical for individuation [6].

## Complexes:

A complex is a cluster of emotionally charged ideas, thoughts and memories that reside in the unconscious and influence behavior and perceptions. Jung linked complexes to archetypes and personal experiences. Complexes can have both positive and negative effects on an individual's psyche [8].

## Psychological types:

Jung proposed a theory of psychological types to explain different patterns of behavior and thought. This classification of people into introverts and extroverts as well as thinking, feeling, sensing and intuiting types. These form the foundation of personality psychology and have influenced modern psychological assessments [6].

## Jung and ETI:

Jung did not directly address the psychiatry of ETI itself. However, Jung discussed strategies with regards to UFO reports. These tactics were primarily from human psychological and symbolic perspectives. Claims of their existence were due to stressors including Cold and Thermo-nuclear War anxiety, hysteria and additional social tensions. Jung further suggested that UFOs might symbolize unknown or alien forces within the human psyche. He considered the possibility that supposed UFO sightings were manifestations of deep, archetypal images rather than evidence of literal extra-terrestrial visitations [9]. Indeed it is important to note that Carl Sagan (1934-1996) was a noted skeptic regarding the existence of UFOs as evidence for ETI. He emphasized the need for extraordinary evidence to support extraordinary claims. Sagan insisted that UFO sightings could be explained by natural or human-made phenomena and he admonished that there was no evidence that UFOs were alien spacecraft [10, 11].

The question of inter-stellar communications and beyond, is complex because it depends on the extent of technological development of the ETI (*e.g.* solving the problem of the cost of high energy throughput), as well as the form that the signaling or communication. On the one hand electromagnetic (light) signals can be occluded over long distances. On the other had the use of neutrinos, which weakly interact with matter, are transmitted across greater distances and durations [12, 13 and 14]. In terms of ETI classifications, the Kardashev Scale was introduced by Kardashev in 1964 and is a hypothetical framework that categorizes civilizations based on their energy consumption and technological developments. Briefly, Type I civilization harnesses all the energy available on its home planet, estimated about 10^16 to 10^17 watts. Type II civilization uses the energy of its star, around 10^26 watts. Type III civilization controls energy at the scale of its galaxy, approximately 10^36 watts. Unfortunately, human civilization is currently below Type I, as our global energy consumption is about 10^13 watts [12, 15].

Table 1 shows a few prior publications that address psychological factors and complications, fundamental in space exploration. These publications did not actually address psychoanalytic consequences and factors. Examples are mentioned in the table with their possible cognate relations with Jung's paradigms. These factors may contribute a basis formulating future potential ETI contact. Such factors may exist for communications among different ETIs.

## Conclusions:

Jung's work is applicable to humans universally. Correspondingly, there are far-reaching questions and comprehensive consequences, which psychiatry broaches with respect to ETI. It is hypothesized here, that Jung's work also impacts the structure, function and development of ETIs. This paper hypothesizes that any ETI with sufficiently developed technology to communicate among stars and galaxies will already have developed the fundamental paradigms and provisions, which Jung promulgated universally for humans. Among paradigms and examples mentioned above, several should be noted and include non-verbal communication, collective unconscious, archetypes, individuation, shadow, anima and animus, synchronicity, self, persona, complexes, psychological types, archetypes, individuation, shadow, anima and animus, synchronicity, self, persona and complexes. These manifest in our speculations about communications with ETI?

The following is a summary of notable concepts that are foundational to Jung's exploration of the human psyche [1, 2]. These terms are possibly fundamental in ETIs. Analogous paradigms and themes are hypothesized to occur in any ETI that has developed long distance communication skills and technology. Humans should be prepared to commence communication with ETIs with these as a basis: Archetypes: Universal symbols or themes appearing in myths, dreams and art across cultures, representing fundamental human experiences, such as the hero or the mother; Collective Unconscious: A shared part of the unconscious mind containing archetypes and memories common to all humans, shaping thoughts and behaviors; Individuation: The psychological process through which a person integrates different aspects of the psyche, achieving personal growth and self-realization; Persona: The social mask or identity that individuals present to the outside world, often differing from their true self; Shadow: The part of the unconscious mind containing repressed weaknesses or desires, often perceived as negative but essential for self-awareness; Anima/Animus: Inner opposite-gender aspects within the psyche; anima represents the feminine qualities in men and animus represents the masculine qualities in women - in this regard irrespective of gender and procreative methods, there would be cross-identification complexities in the ETI psychology; Synchronicity: The concept of meaningful coincidences that aren't causally related, suggesting an interconnectedness of all events; Self: The central archetype of wholeness and the ultimate goal of individuation, integrating conscious and unconscious aspects of the personality; Introversion/Extraversion: Personality orientations where introverts focus on internal thoughts and feelings and extraverts focus on the external environment; Complexes: Emotionally charged groups of ideas or experiences in the unconscious, often influencing behavior. The role of Jung's theories suggests that humanity may project certain unconscious and conscious desires or fears onto the awareness of ETI civilizations, which could ruin potential constructive interactions with ETIs.

## Figures and Tables

**Table 1:** Psychological factors and Jung paradigms

**Reference**	**Summary**	**Jung Consistency, Categories and Analysis**
[16]	Harrison explores the intersection of science, religion and folklore through the lens of cosmic visions. He analyzes various cultural interpretations of extraterrestrial phenomena and their implications for human understanding of the universe.	Archetypes, Collective unconscious, Universal symbols, Myths related to cosmic predictions. The exploration of cosmic visions aligns with Jung's ideas of collective symbols, reflecting how different cultures interpret the unknown, facilitating individuation.
[17]	Kottmeyer examines the psychological dimensions of potential alien encounters, positing that these experiences can be understood as manifestations of the collective unconscious. He discusses the symbolic nature of UFO sightings and abduction scenarios as reflections of deeper human anxieties and archetypal motifs.	Collective unconscious, Archetypes, Alien encounters could symbolize deeper psychological themes. A possible alien encounter as manifestations of the collective unconscious reflects Jung's theories on how our psyche interacts with external symbols, revealing inner anxieties.
[18]	Lemarchand provides a theoretical framework for understanding the evolution of extraterrestrial intelligence (ETI) within the context of cosmic evolution. He integrates astrophysics and biological evolution to discuss potential forms and pathways of ETI development.	Self and Individuation through the exploration of humanity's place in the cosmos, though it is more scientific than psychological. This theoretical exploration invites reflection on the human psyche's evolution, akin to Jung's notion of individuation, as we seek to understand our possible role in a broader cosmic context.
[19]	Persinger introduces the concept of quantum resonance as a potential mechanism for non-verbal communication with extraterrestrial intelligences. He explores the implications of such interactions for the psyche.	Limited connection to Jungian analysis; communication and connection, Jung's focus on interpersonal relationships and Non-verbal communication opens discussions about the synchronicity and collective experiences that may align with Jung's ideas on the interconnectedness of all beings.synchronicity.
[20]	Shostak shares his experiences and insights as a leading scientist in the search for extraterrestrial intelligence. The book discusses the scientific methods employed in the search and reflects on the cultural and philosophical implications of discovering ETI.	Jung's exploration of the psyche, Symbolic representations, Search for ETI can reflect humanity's quest for understanding the Individuation process, Search for ETI mirrors our quest for self-understanding.unknown.
[21]	Todd and Miller investigate whether evolutionary psychology can provide insights into the behaviors and adaptations related to ETI. They analyze potential universal patterns in search, signaling and aversion among messaging ETIs.	Archetypes in terms of universal patterns of behavior, Evolutionary mechanisms, psychological themes. Search, signaling and aversion, ETI may reveal archetypal behaviors that resonate with Jungian concepts of instinctual drives and shared human experiences.
[22]	Vakoch examines the concept of altruism in the context of extraterrestrial life, discussing the ethical implications of potential interactions between humans and ETI. He explores evolutionary theories that could explain altruistic behaviors in the cosmos.	Directly relates to Jungian ethics and the concept of altruism, which is significant in personal and collective growth within Jung's framework of psychological development. Altruism invites reflection on ethical dimensions in human interactions, aligning with Jung's ideas of compassion and interconnectedness in the evolution of consciousness. This is the antithesis of Thucydides' concept of hiding and defense. [23]
[24]	Psychological aspects of space exploration, historical perspectives. Psychological challenges faced by astronauts.	Individuation and the Collective psyche. Space exploration seen as a metaphor for self-discovery and understanding one's place in the universe. Individuation process, self-understanding, the integration of the collective psyche as self-identities.
